# Supplementary material for: A robust approach for MicroED sample preparation of lipidic cubic phase embedded membrane protein crystals
Source: Nat Commun. 2023 Feb 25;14:1086. doi: 10.1038/s41467-023-36733-4 (PMC9968316; doi:10.1038/s41467-023-36733-4)
Supplement: Supplementary file 1 — Supplementary Information [file 41467_2023_36733_MOESM1_ESM.pdf]

Supplementary Information

A robust approach for MicroED sample preparation of lipidic cubic phase embedded membrane protein crystals

Michael W. Martynowycz[1,2], Anna Shiriaeva[1,2], Max T. B. Clabbers[1,2], William J. Nicolas[1,2], Sara J. Weaver[1,2], Johan Hattne[1,2], Tamir Gonen[1,2,3$]

[1] Howard Hughes Medical Institute, University of California, Los Angeles CA 90095

[2] Department of Biological Chemistry, University of California, Los Angeles CA 90095

[3] Department of Physiology, University of California, Los Angeles CA 90095

[$] Correspondence: tgonen@g.ucla.edu

List of supplementary items

Supplementary Fig. 1. Milling LCP using a gallium ion beam.

Supplementary Fig. 2. SRIM calculations for pFIB milling vitreous material

Supplementary Fig. 3. Correlation between the iFLM and pFIB measured depth

Supplementary Fig. 4. Grazing incidence GIS platinum deposition aided by the plasma ion beam

Supplementary Fig. 5. SEM and pFIB images of proteinase crystals milled using the xenon beam

Supplementary Fig. 6. SEM and pFIB images of proteinase crystals milled using the argon beam

Supplementary Fig. 7. SEM and pFIB images of proteinase crystals milled using the nitrogen beam

Supplementary Fig. 8. SEM and pFIB images of proteinase crystals milled using the oxygen beam

Supplementary Fig. 9: TEM images of proteinase crystals milled using the xenon beam

Supplementary Fig. 10. TEM images of proteinase crystals milled using the argon beam

Supplementary Fig. 11. TEM images of proteinase crystals milled using the nitrogen beam

Supplementary Fig. 12. TEM images of proteinase crystals milled using the oxygen beam

Supplementary Fig. 13. Crystallographic statistics for xenon ion-beam milled lamellae

Supplementary Fig. 14. Crystallographic statistics for argon ion-beam milled lamellae

Supplementary Fig. 15. Crystallographic statistics for nitrogen ion-beam milled lamellae

Supplementary Fig. 16. Crystallographic statistics for oxygen ion-beam milled lamellae

Supplementary Fig. 17. Crystallographic statistics for best merge from all ion-beam milled lamellae

Supplementary Fig. 18. Targeting of labelled protein crystals.

Supplementary Fig. 19. Correlation of iFLM and SEM/FIB using 3DCT.

Supplementary Table 1. Milling steps for each plasma ion beam experiment on proteinase K

Supplementary Table 2. Milling steps for each plasma ion beam experiment on $A_{2A}AR$

Supplementary Table 3. Milling currents for each available ion source on the pFIB

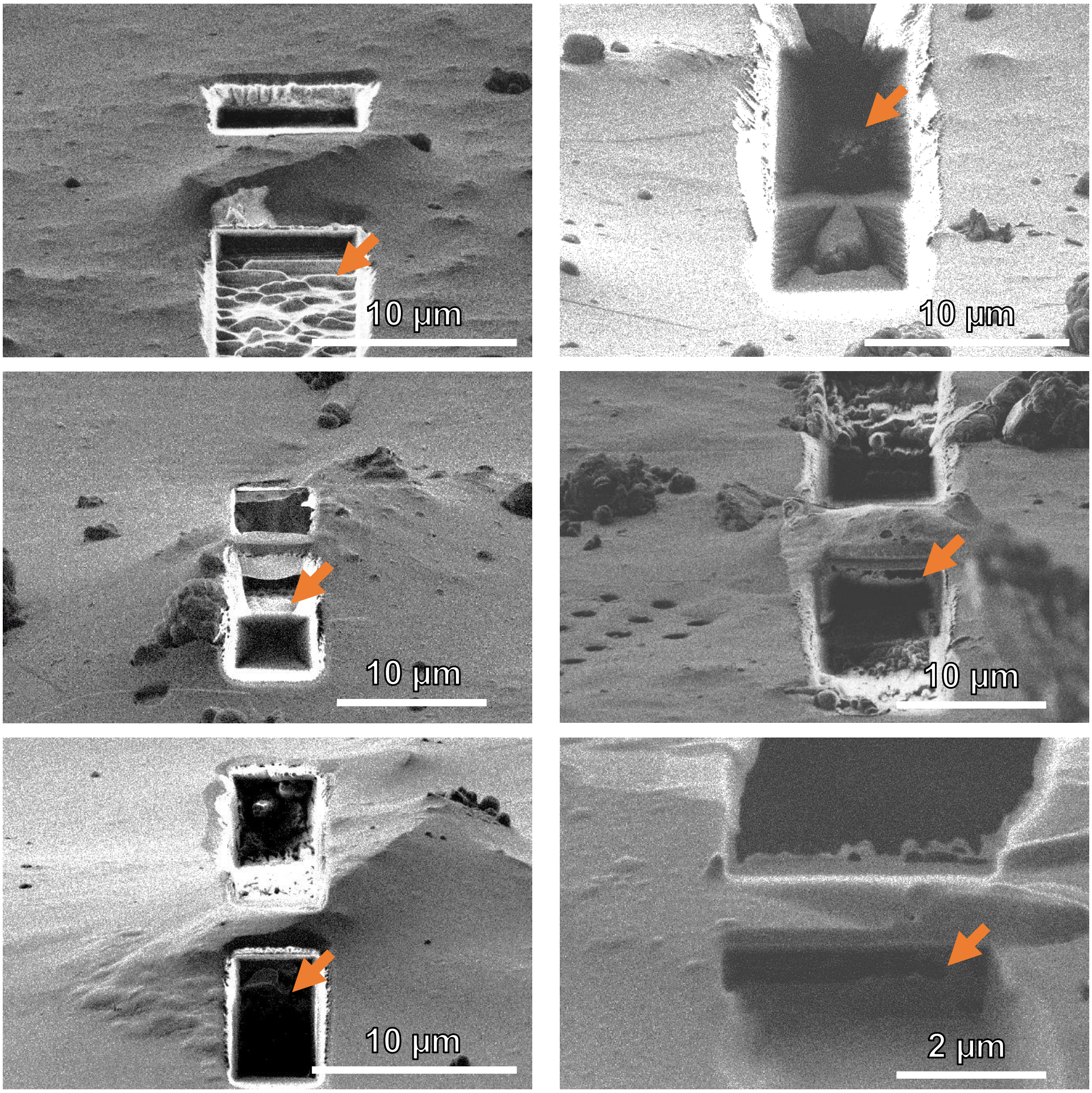

Supplementary Fig. 1. Milling LCP using a Gallium ion beam. Several examples are given showing various pathologies after attempted milling using a liquid metal FIB. Orange arrows point to various issues encountered during milling. These pathologies persisted after up to one hour of milling at currents reaching 1 nA, where the underlying lamellae were no longer viable due to damage.

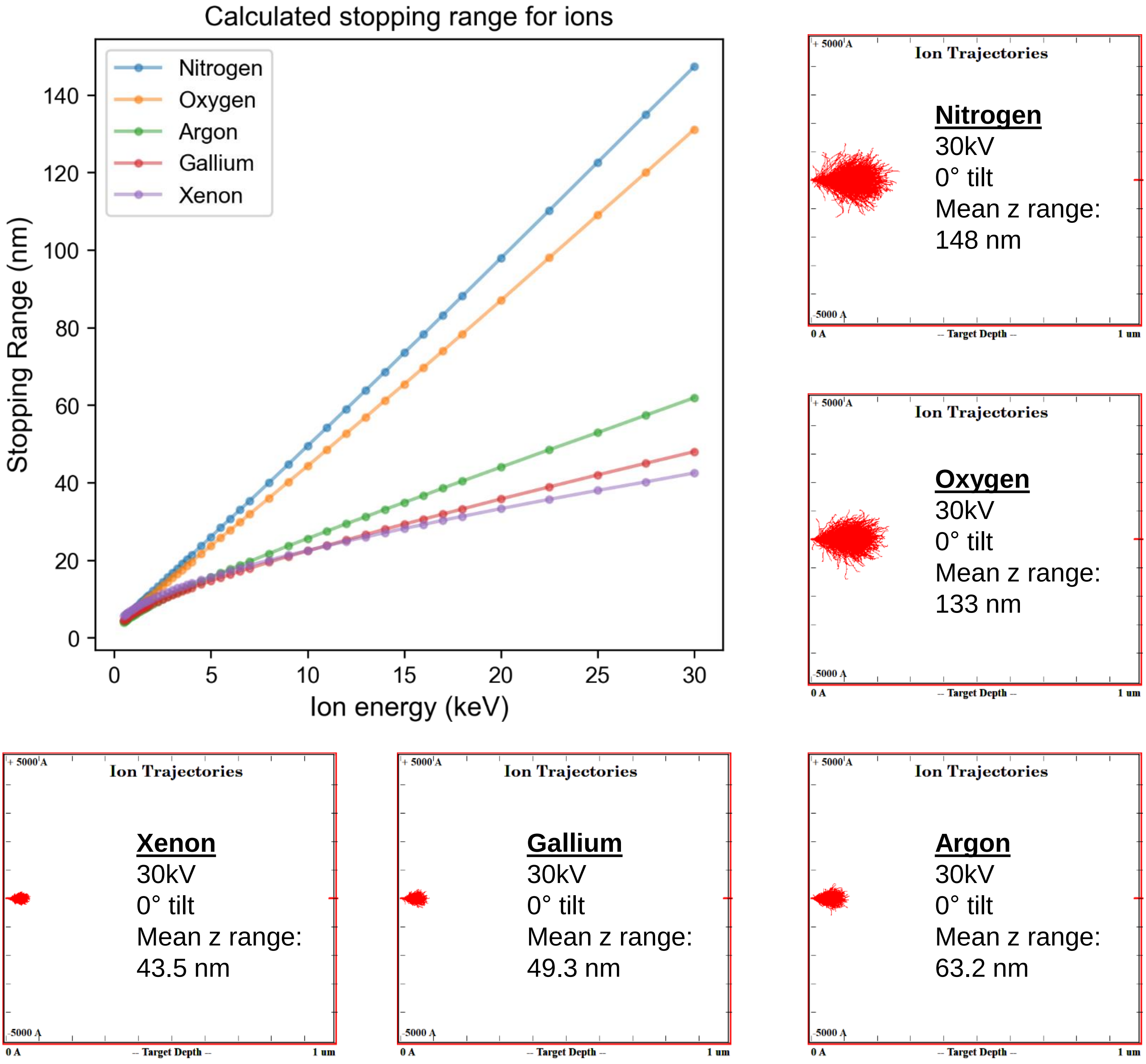

Supplementary Fig. 2. SRIM calculations for pFIB milling vitreous material. The tabulated values for the various ion species are plotted as a function of accelerating voltage. Individual panels show the mean range in vitreous ice for each ion species calculated using SRIM2018. Simulations show average over 1000 interactions.

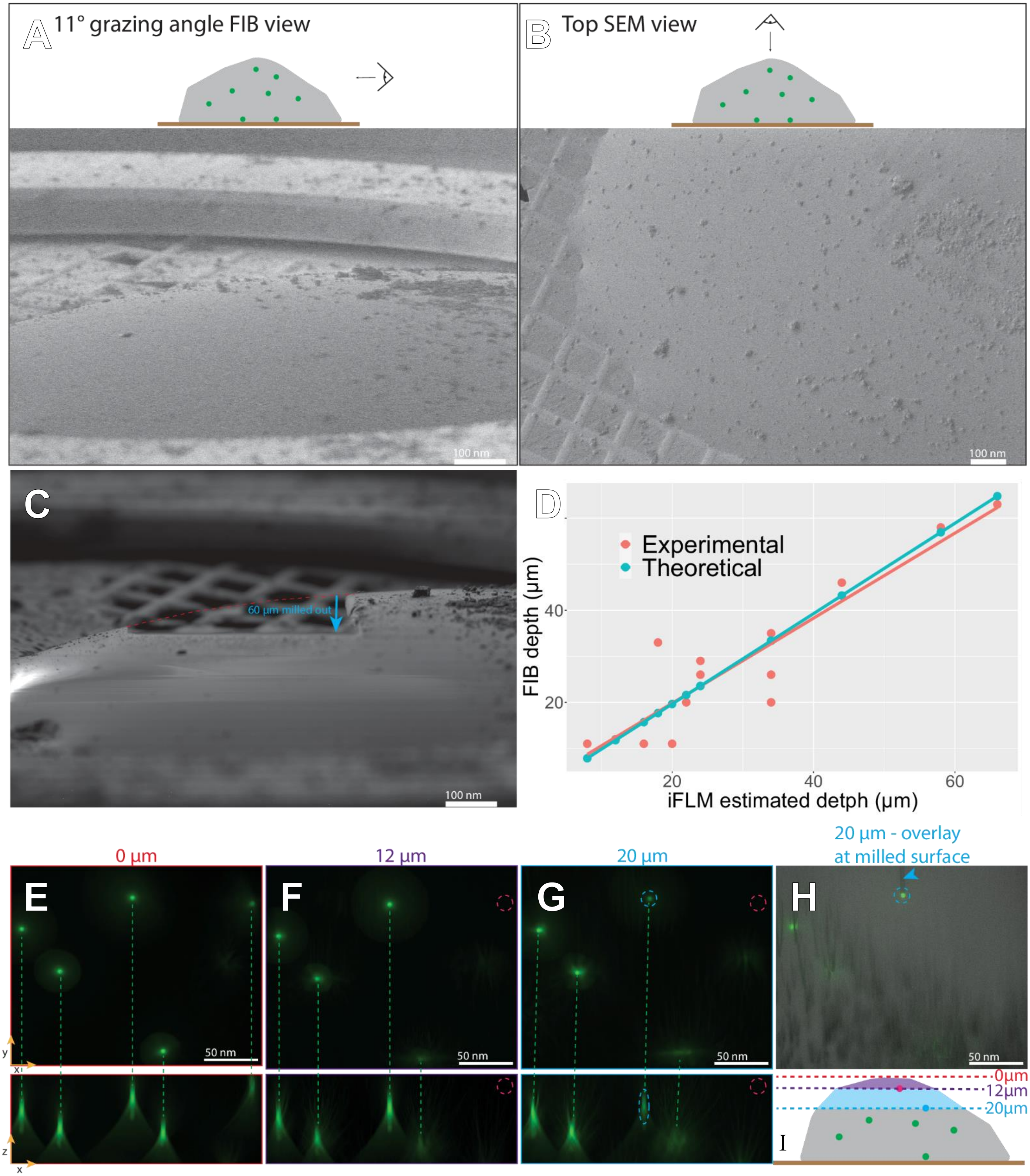

Supplementary Fig. 3. Caption on following page.

Supplementary Fig 3. Correlation between the iFLM and pFIB measured depth. (**A**) Top panel: Cartoon of a glycerol pile (gray) laying on a grid (orange) with 4 um Tetraspecs encased in it (green). The eye and arrow represent the grazing angle at which the imaging/milling is done with the FIB gun. Bottom panel: FIB view using Argon at 6pA – 30kV showing the frozen glycerol pile. (**B**) Top panel: Same cartoon as in (A) with the eye and arrow representing the top view acquired from the electron gun. Bottom panel: SEM view at 25pA – 500V of the pile of glycerol. (**C**) FIB view at 4nA – 30kV and same angle as in (A) after multiple incremental milling steps. Here, 60 µm have been milled in total (blue arrow). The red outline shows the initial curve of the glycerol pile. (D) Plot of the iFLM measured depth (x-axis) versus the FIB measured depth (y-axis). (**E, F and G**) Top panel: Maximum intensity projection of an X-Y oriented stack acquired at the milling site. Bottom panel: Maximum intensity projection of an X-Z oriented stack acquired at the milling site. (E) was acquired before any milling was performed. It is the zero reference. (F and G) were acquired after milling 12 µm and 20 µm, respectively. The dashed purple circles show a Tetraspec that disappeared after 12 µm of milling. The yellow dashed purple circles represent a Tetraspec that is spot-on 20 µm deep. (**H**) Overlay optical slice at 20 µm deep showing the surface of the lamella in reflective mode (gray) and the fluorescent Tetraspecs (green). The bead circled in blue sits on the milled surface, its milled "shadow", creating a curtaining artefact can be seen behind it (blue arrowhead). (**I**) Same cartoon as in (A) and (B) with dashed lines corresponding to the different depths at which the iFLM stacks showcased in (E, F and G) were acquired.

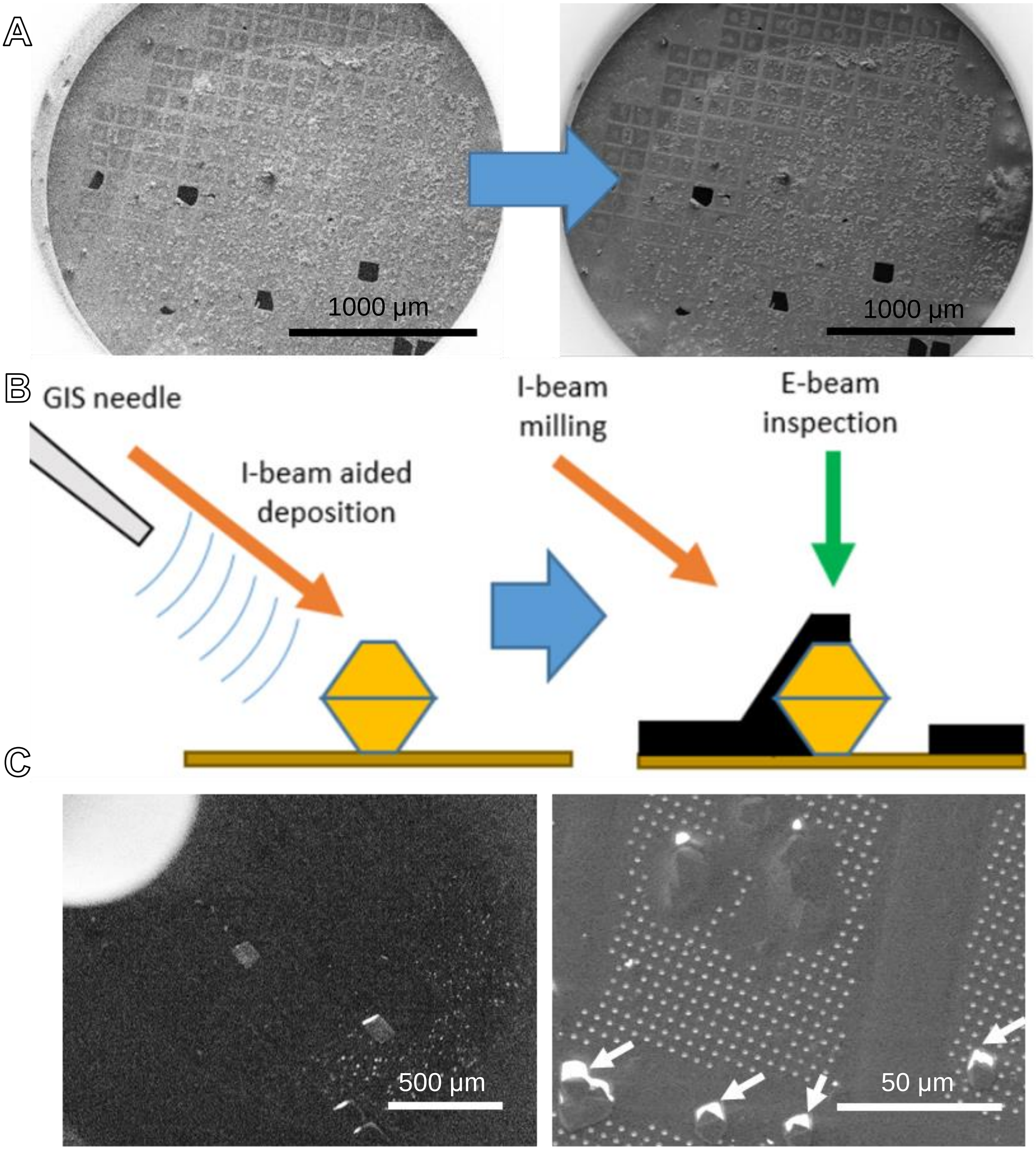

Supplementary Fig. 4. Grazing incidence GIS platinum deposition aided by the plasma ion beam. (A) whole-grid SEM image before (left) and after (right) GIS deposition. (B) Cartoon depiction of ion-assisted GIS platinum deposition (left) and how the geometry leaves the back of the crystal shadowed (right). (C) The left-hand image shows the view in the xenon ion-beam during the GIS platinum coating of the grid, and the right-hand side shows the grid after coating with clear uncoated areas behind each crystal.

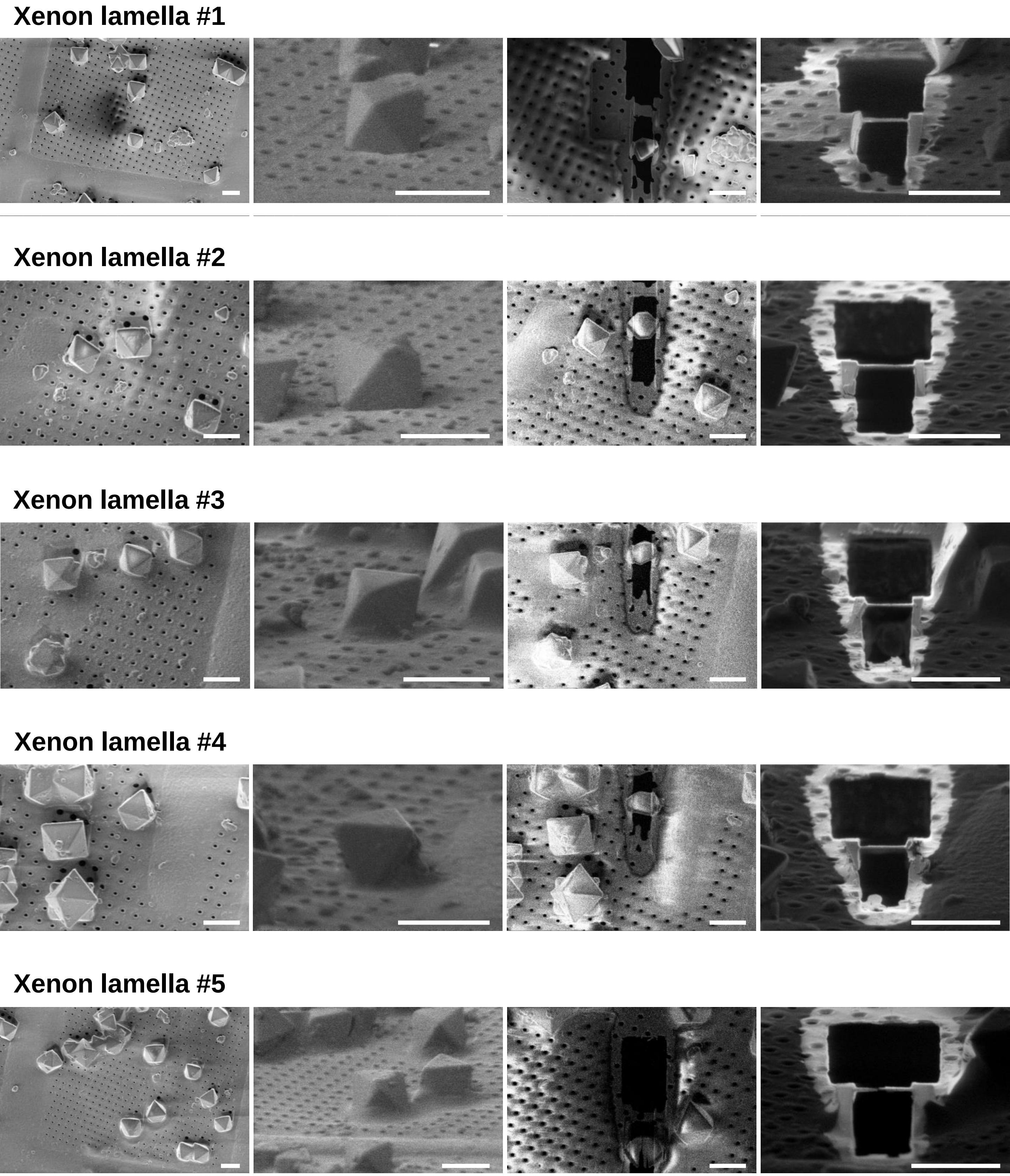

Supplementary Fig. 5. SEM and pFIB images of proteinase crystals milled using the xenon beam. All scale bars are 10 µm.

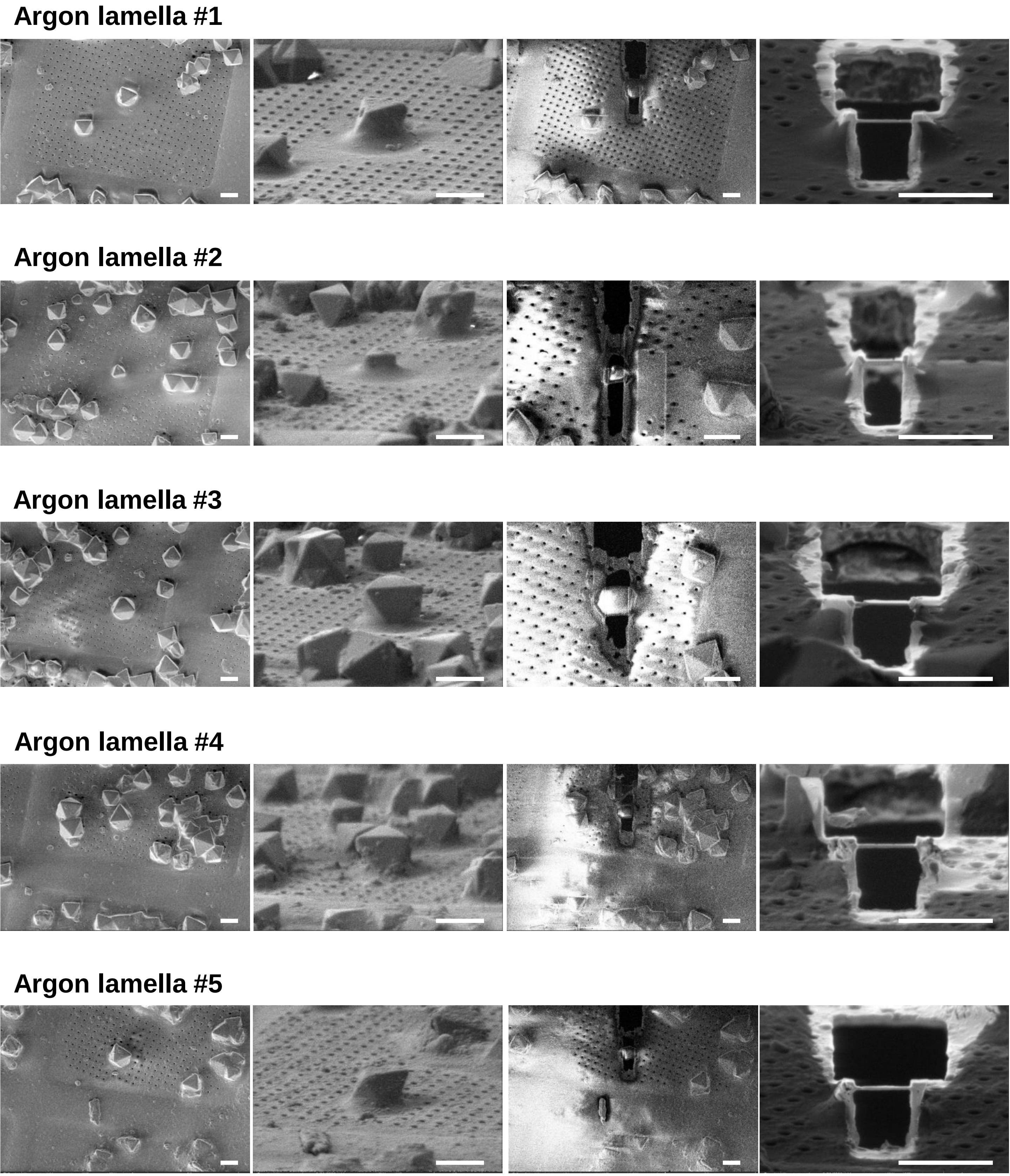

Supplementary Fig. 6. SEM and pFIB images of proteinase crystals milled using the argon beam. All scale bars are 10 µm.

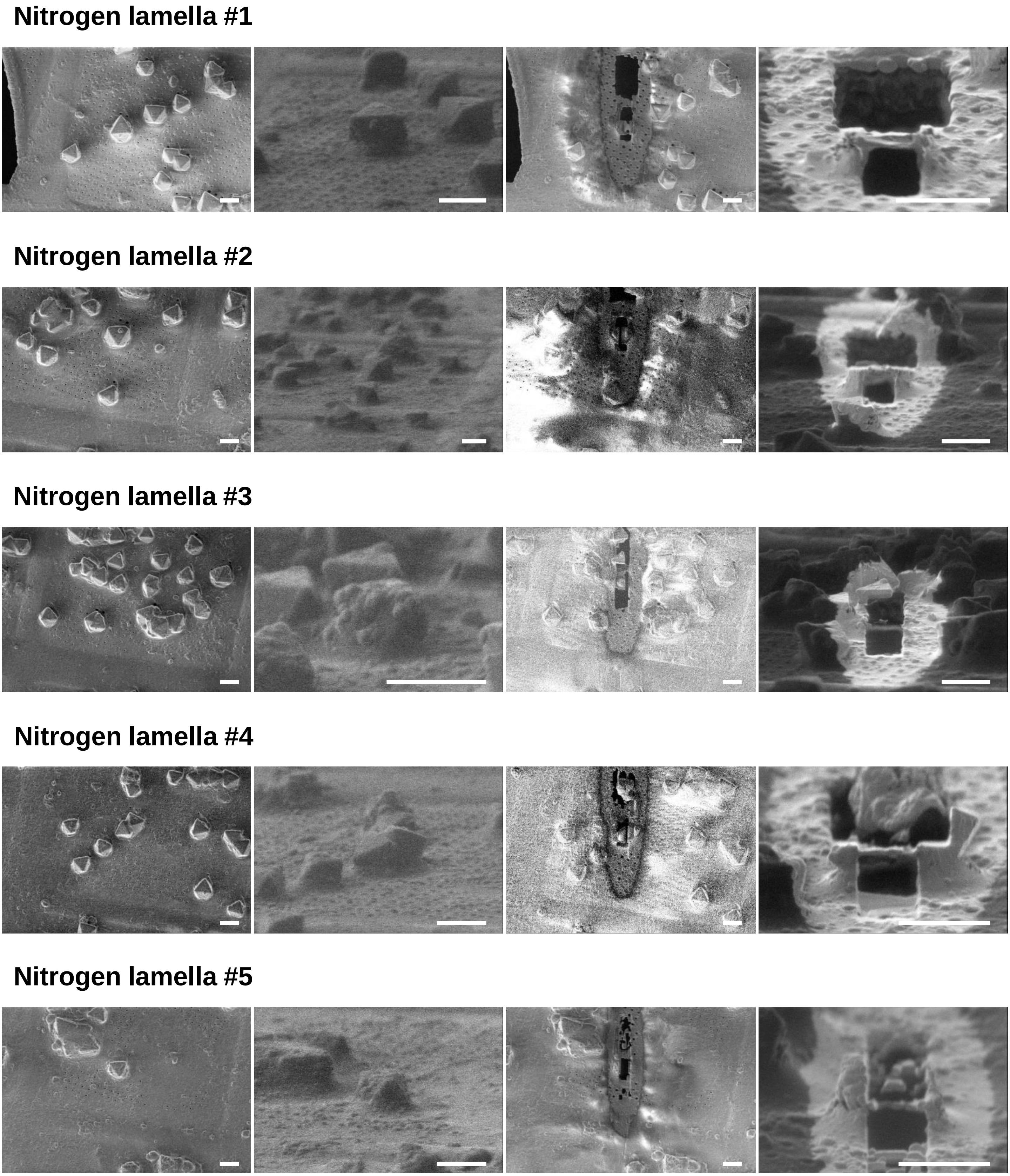

Supplementary Fig. 7. SEM and pFIB images of proteinase crystals milled using the nitrogen beam. All scale bars are 10 µm.

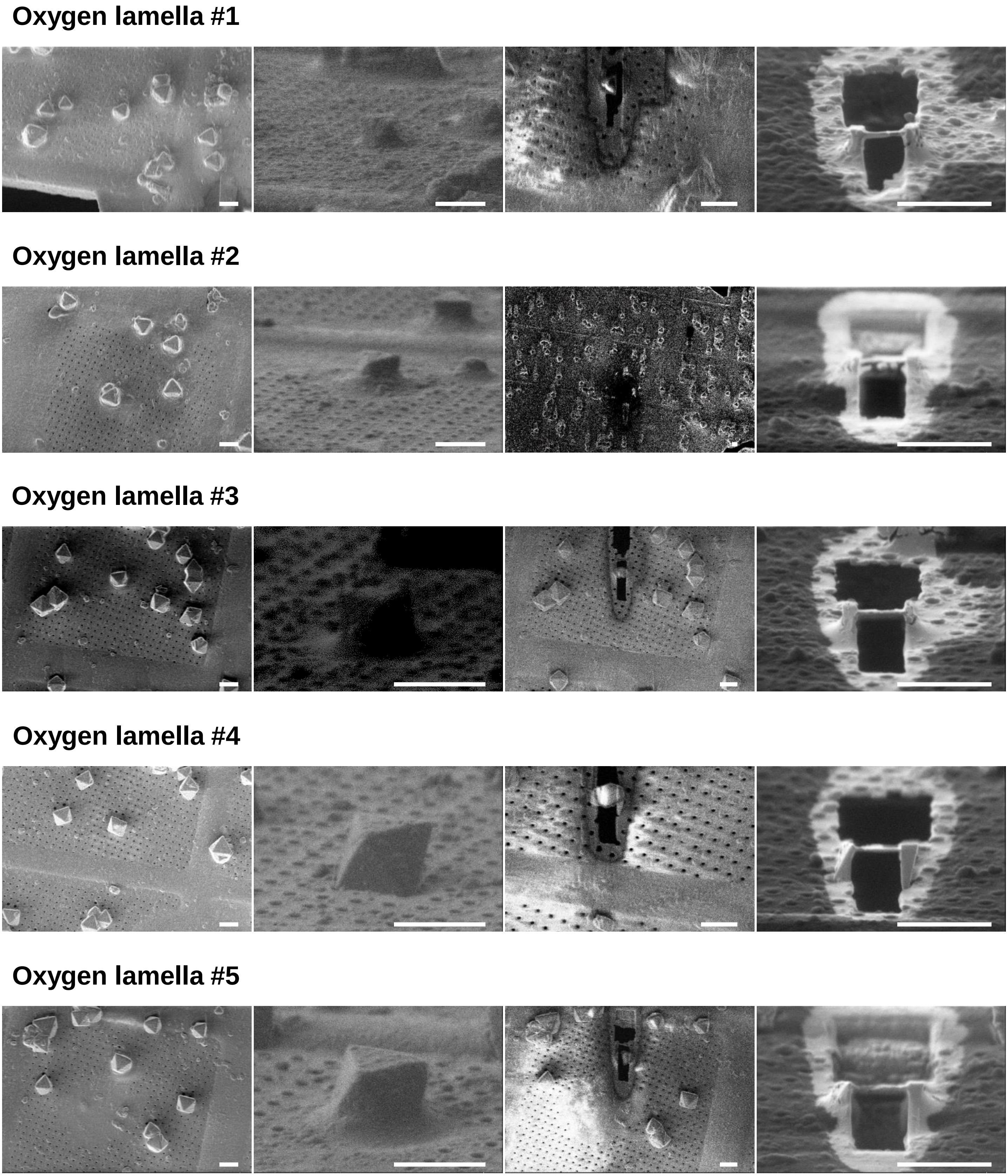

Supplementary Fig. 8. SEM and pFIB images of proteinase crystals milled using the oxygen beam. All scale bars are 10 µm.

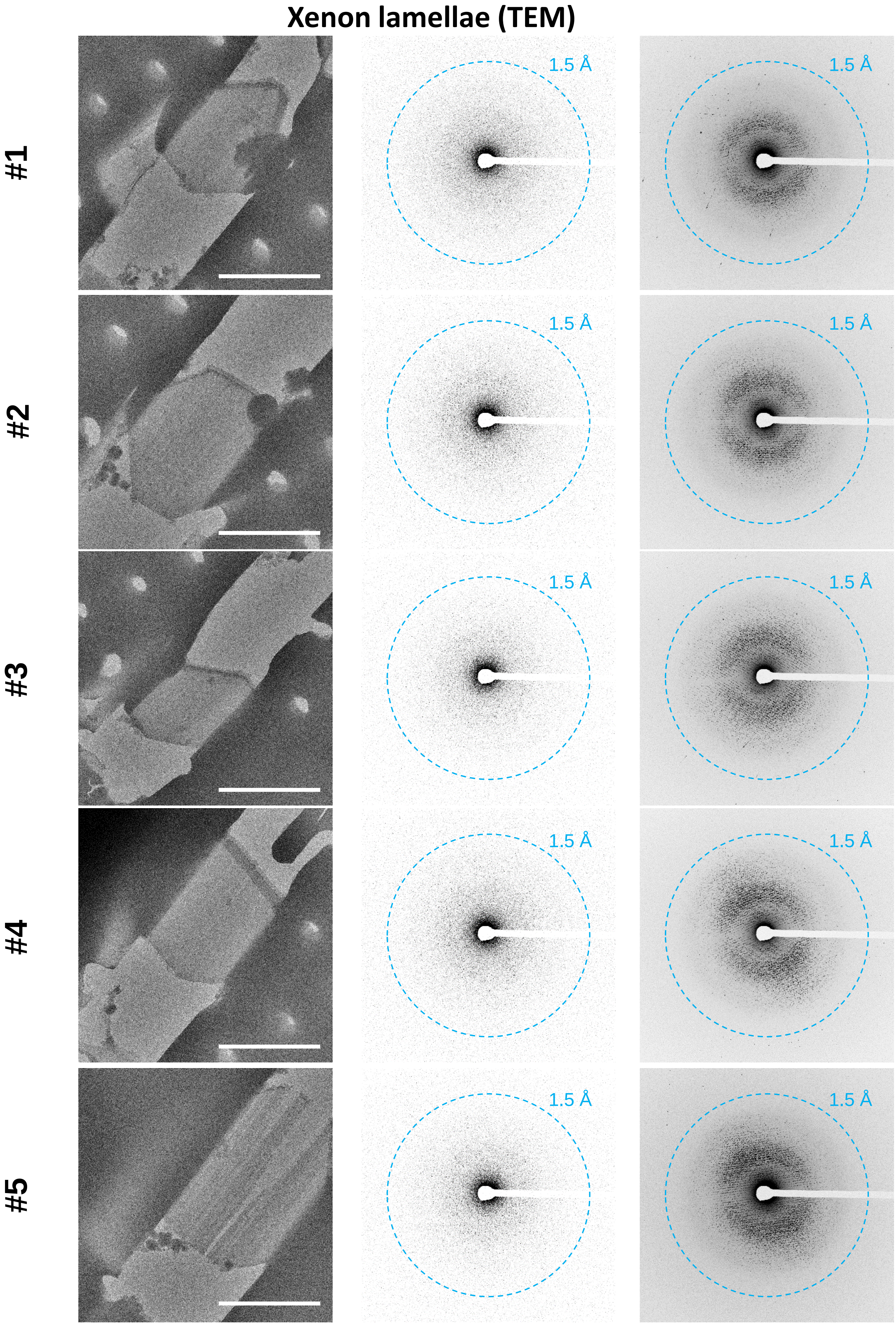

Supplementary Fig. 9. Caption appears on following page.

Supplementary Fig. 9. TEM images of proteinase crystals milled using the xenon beam. All scale bars are 10 µm. Single diffraction images from each movie are depicted in the center, and the maximum intensity projections shown on the right.

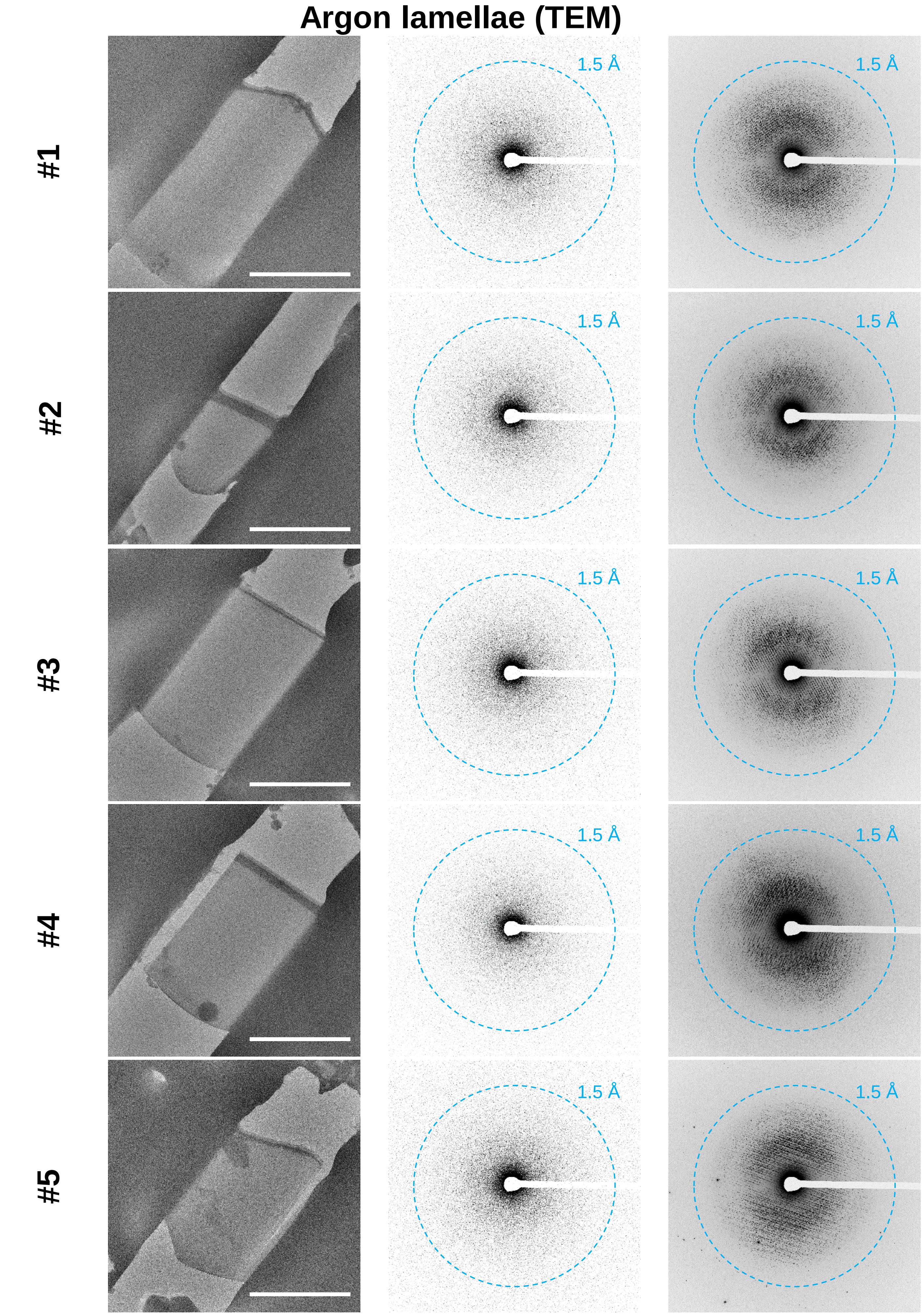

Supplementary Fig. 10. Caption appears on following page.

Supplementary Fig. 10. TEM images of proteinase crystals milled using the argon beam. All scale bars are 10 µm. Single diffraction images from each movie are depicted in the center, and the maximum intensity projections shown on the right.

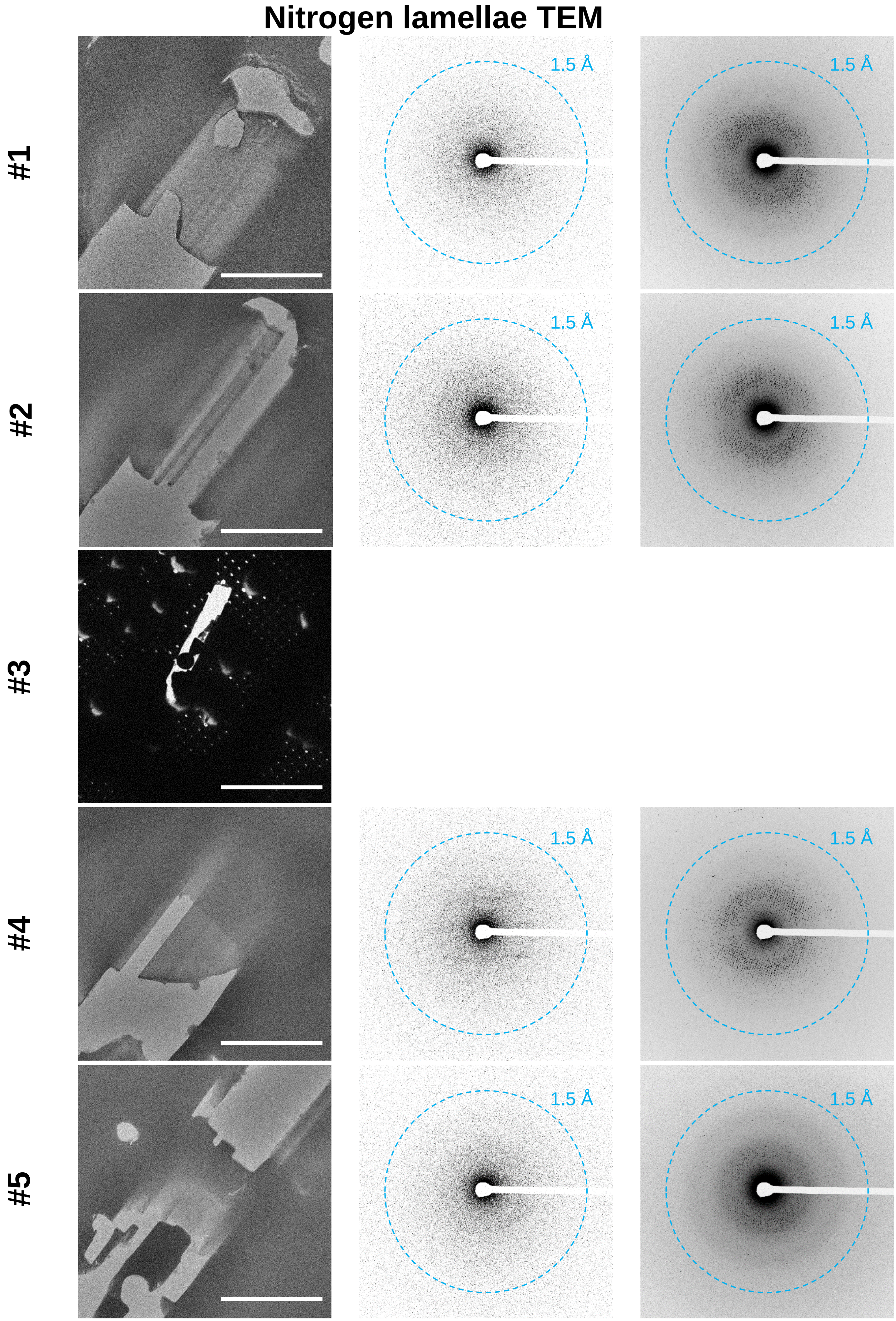

Supplementary Fig. 11. Caption appears on following page.

Supplementary Fig. 11. TEM images of proteinase crystals milled using the nitrogen beam. All scale bars are 10 µm. Single diffraction images from each movie are depicted in the center, and the maximum intensity projections shown on the right.

Oxygen lamellae TEM

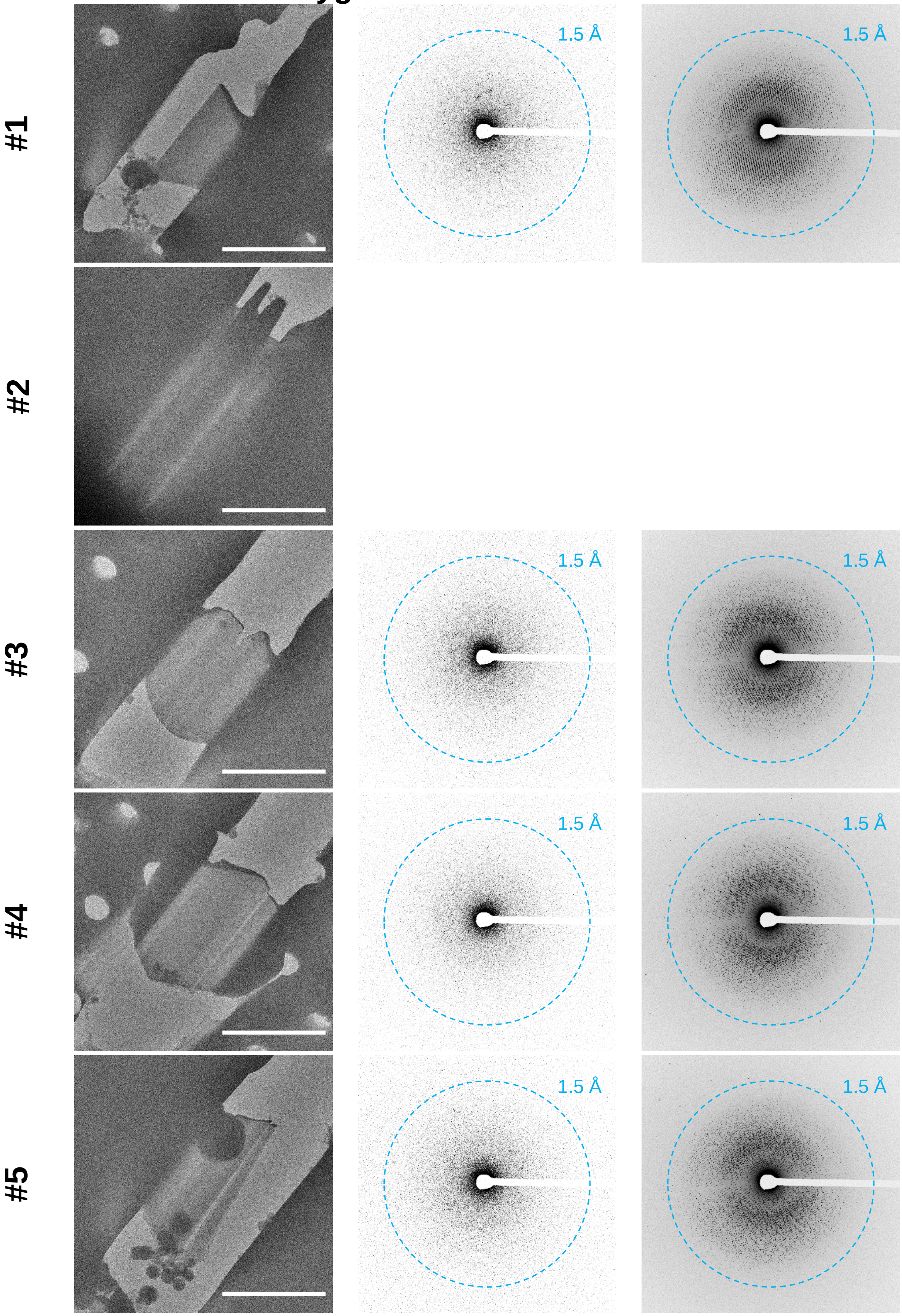

Supplementary Fig. 12. Caption appears on following page.

Supplementary Fig. 12. TEM images of proteinase crystals milled using the oxygen beam. All scale bars are 10 µm. Single diffraction images from each movie are depicted in the center, and the maximum intensity projections shown on the right.

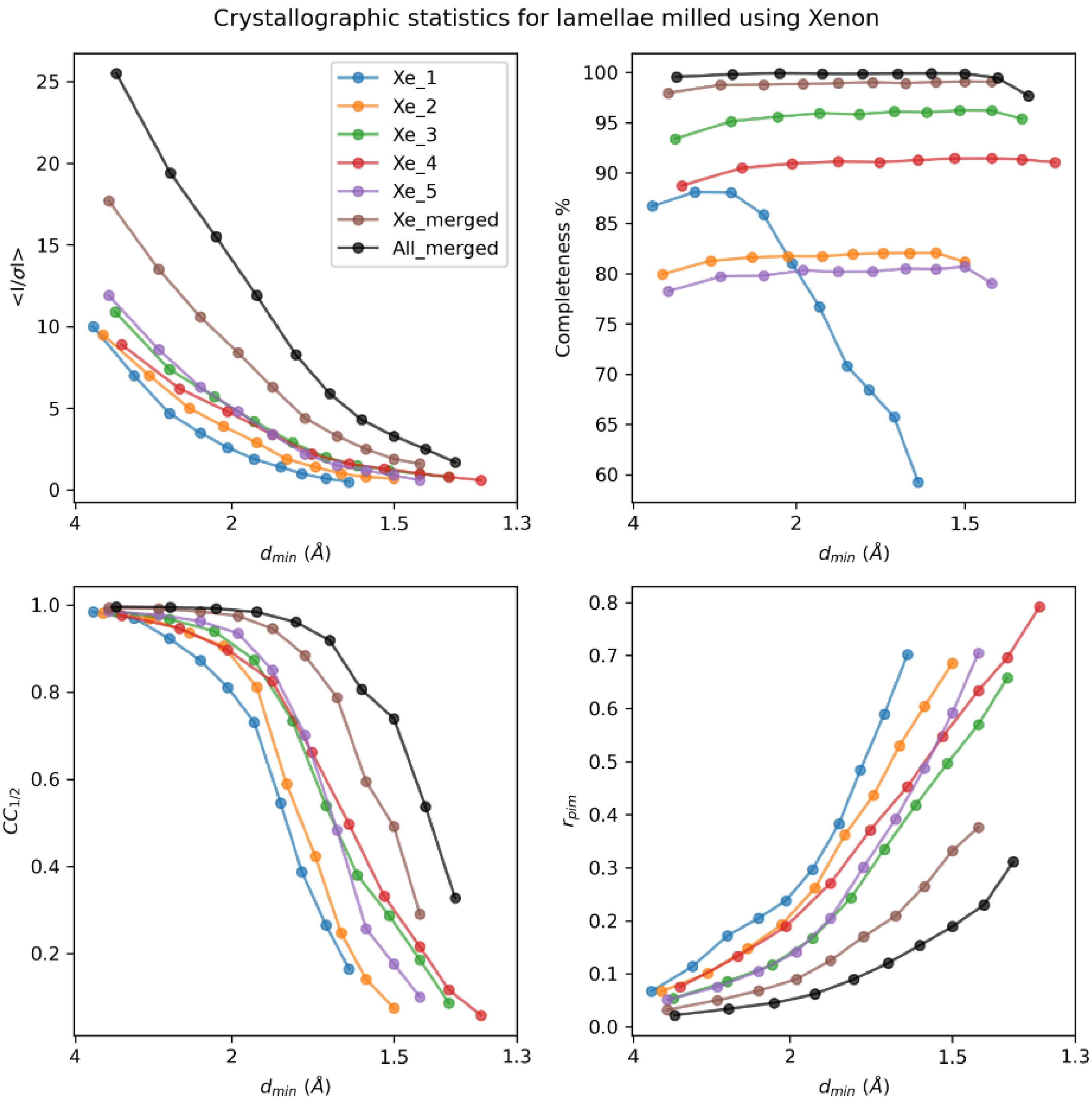

Supplementary Fig. 13. Crystallographic statistics for xenon ion-beam milled lamellae. Plots depict the mean signal to noise ratio (<I / σ (I)>) (top left), completeness (%) (top right), mean half-set correlation coefficient ($CC_{1/2}$) (bottom left), and multiplicity corrected R factor (R_{pim})(bottom right) as functions of the d_{min} resolution bins (Å). The “best merge” data set is included for comparison in each case.

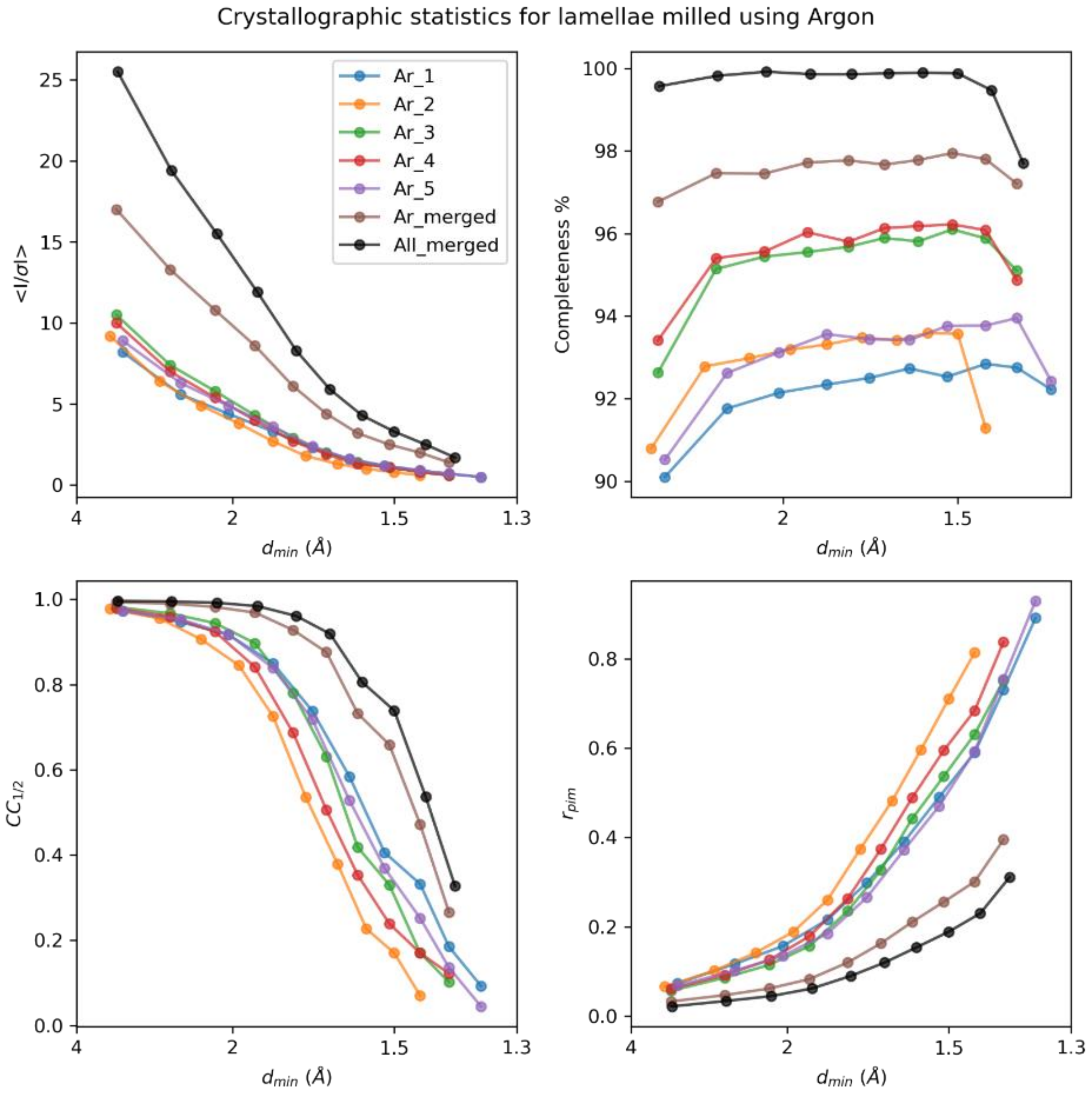

Supplementary Fig. 14. Crystallographic statistics for argon ion-beam milled lamellae. Plots depict the mean signal to noise ratio (<I / σ (I)>) (top left), completeness (%) (top right), mean half-set correlation coefficient ($CC_{1/2}$) (bottom left), and multiplicity corrected R factor (R_{pim})(bottom right) as functions of the d_{min} resolution bins (Å). The "best merge" data set is included for comparison in each case.

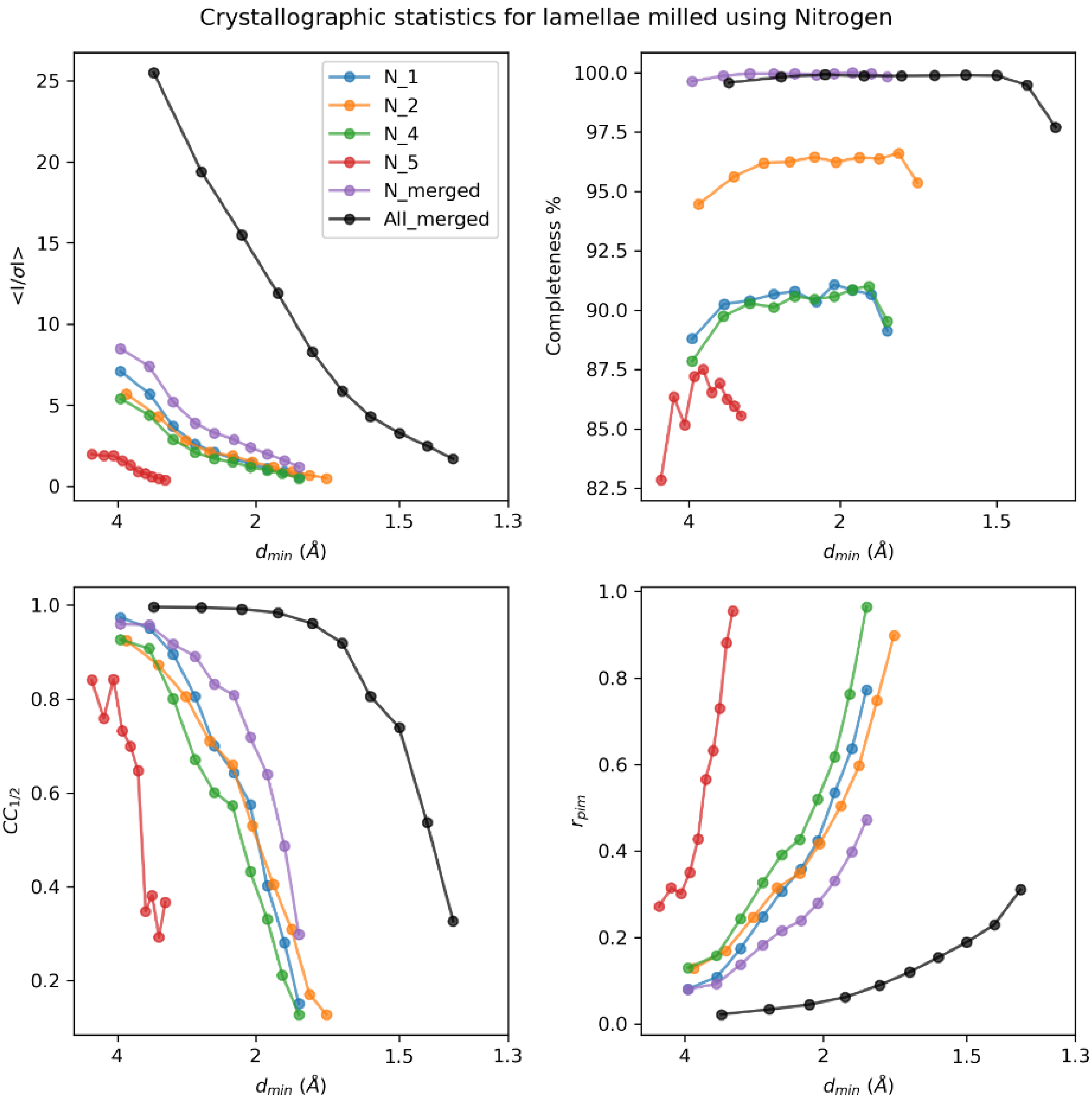

Supplementary Fig. 15. Crystallographic statistics for nitrogen ion-beam milled lamellae. Plots depict the mean signal to noise ratio (<I / σ (I)>) (top left), completeness (%) (top right), mean half-set correlation coefficient ($CC_{1/2}$) (bottom left), and multiplicity corrected R factor (R_{pim})(bottom right) as functions of the d_{min} resolution bins (Å). The “best merge” data set is included for comparison in each case.

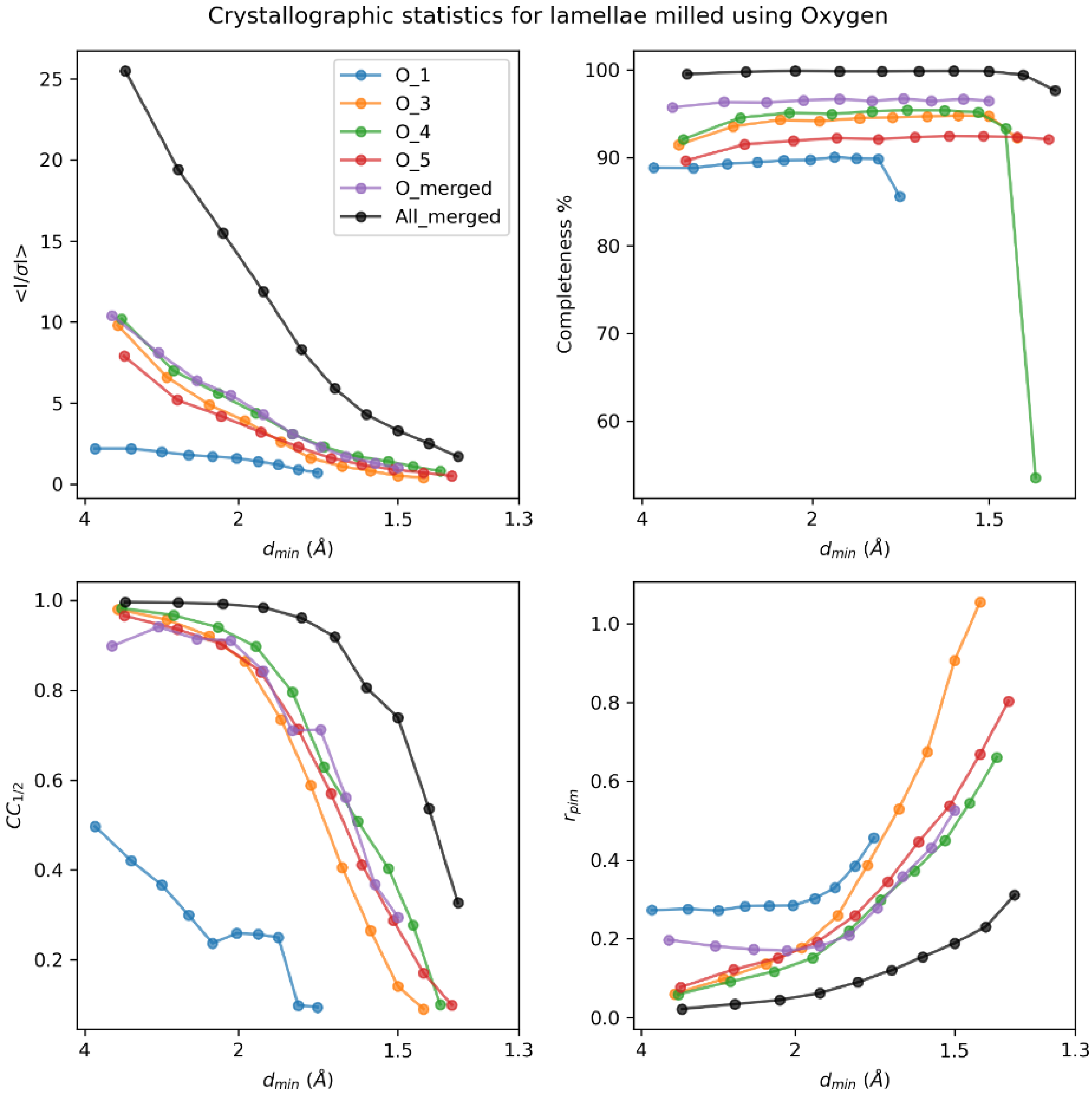

Supplementary Fig. 16. Crystallographic statistics for oxygen ion-beam milled lamellae. Plots depict the mean signal to noise ratio (<I / σ (I)>) (top left), completeness (%) (top right), mean half-set correlation coefficient ($CC_{1/2}$) (bottom left), and multiplicity corrected R factor (R_{pim})(bottom right) as functions of the d_{min} resolution bins (Å). The “best merge” data set is included for comparison in each case.

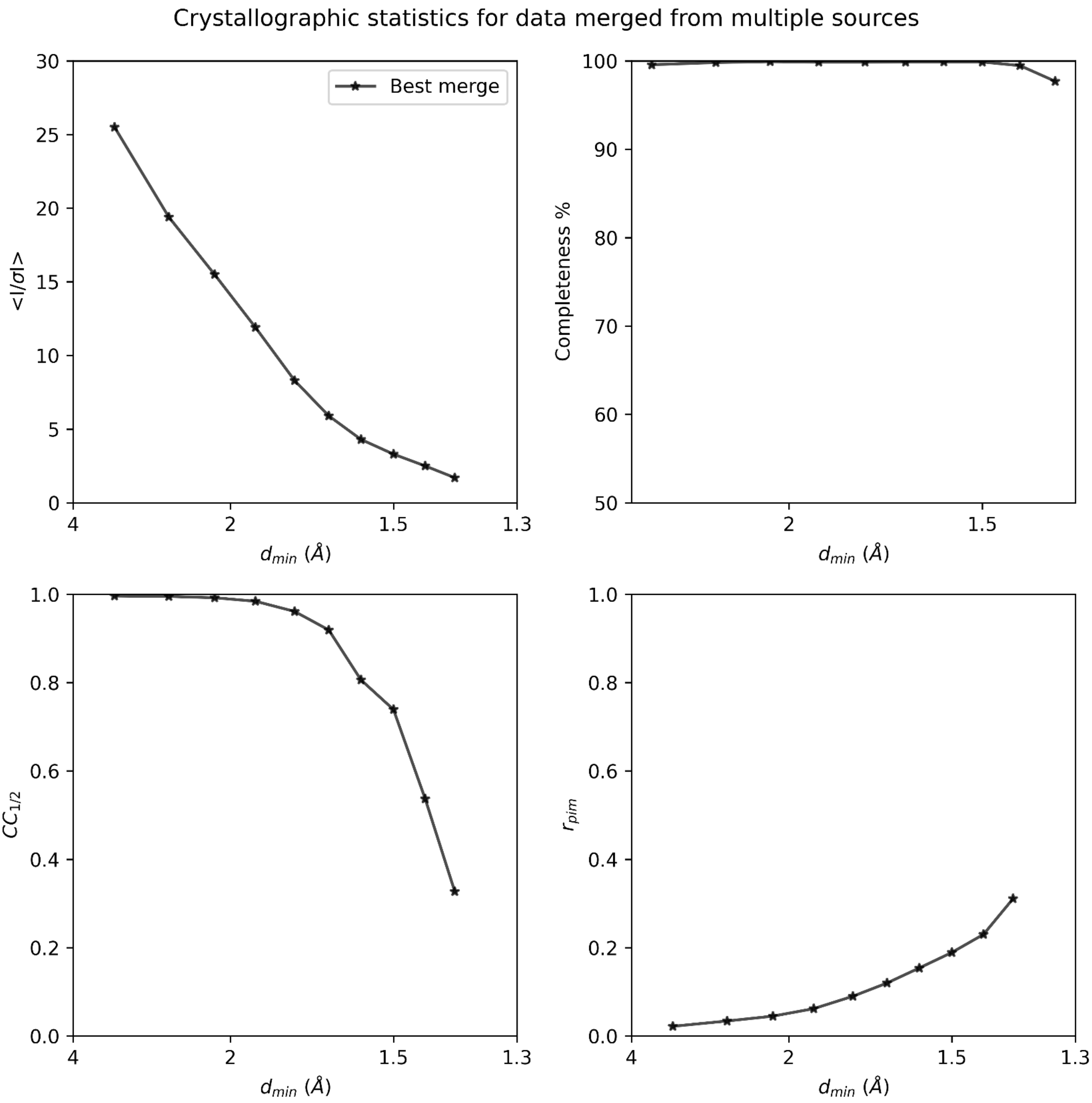

Supplementary Fig. 17. Crystallographic statistics for best merge from all ion-beam milled lamellae. Plots depict the mean signal to noise ratio (<I / σ (I)>) (top left), completeness (%) (top right), mean half-set correlation coefficient ($CC_{1/2}$) (bottom left), and multiplicity corrected R factor (R_{pim})(bottom right) as functions of the d_{min} resolution bins (Å).

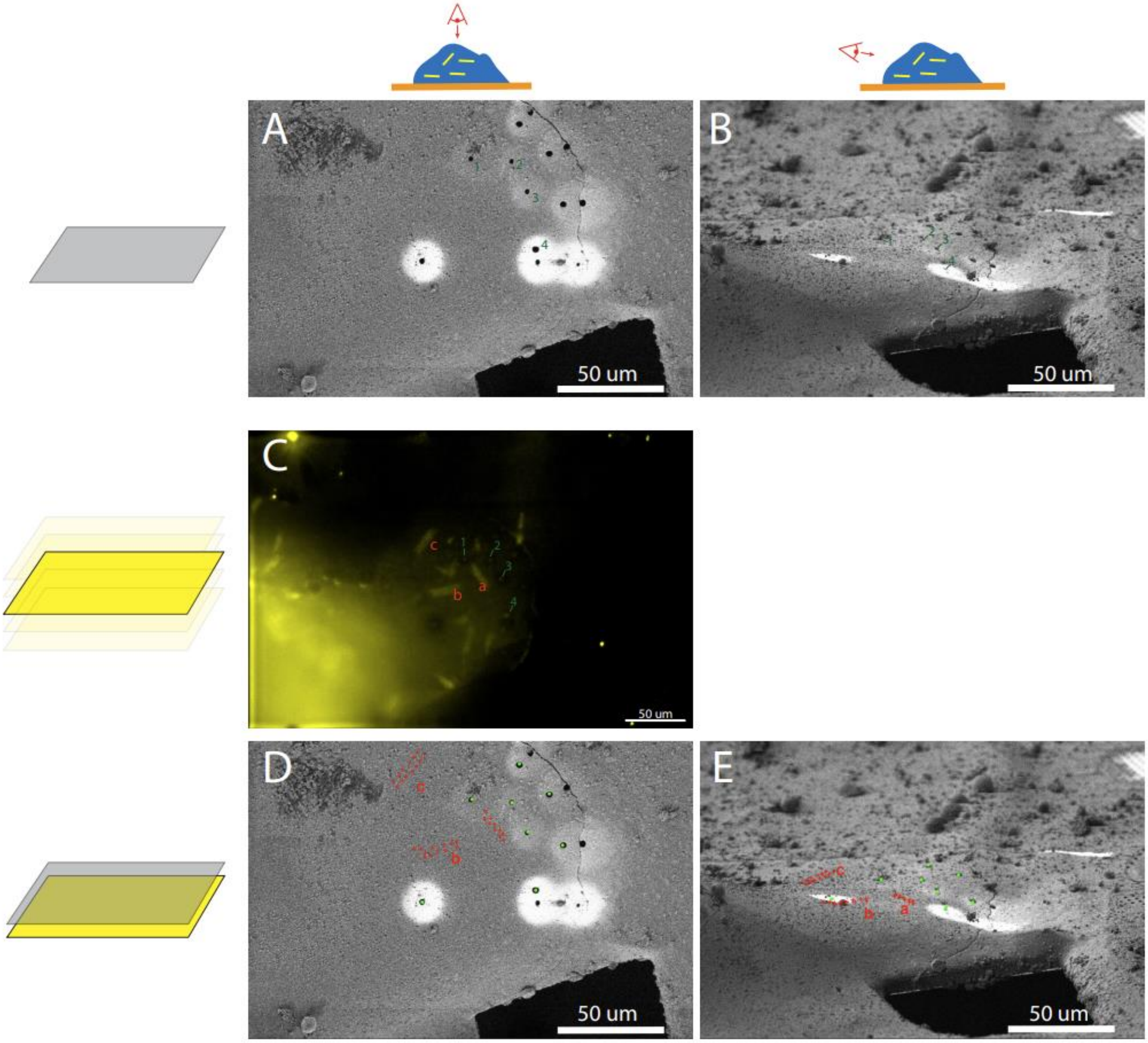

Supplementary Fig. 18. (A) 90° top-down FIB view of milling area. Four milled fiducials are designated with green numbers. (B) 23° grazing-angle FIB view of the same milling area as in (A). The same four milled fiducials are also designated with green numbers. (C) Optical slice through a deconvolved iFLM volume showing 3 crystals (labelled in red “a”, “b” and “c”). The same four milled fiducials designated in (A) and (B) are shown in green. (D) Correlated 90° top-down FIB view shown in (A) and the fluorescent stack shown in (C). Red crosshairs show where the “a”, “b” and “c” crystals of interest are predicted, and the milled fiducials used to do the correlation are in green. (E) Correlated 23° grazing-angle FIB view shown in (B) and the fluorescent stack shown in (C). Red crosshairs show where the “a”, “b” and “c” crystals of interest are predicted, and the milled fiducials used to do the correlation are in green.

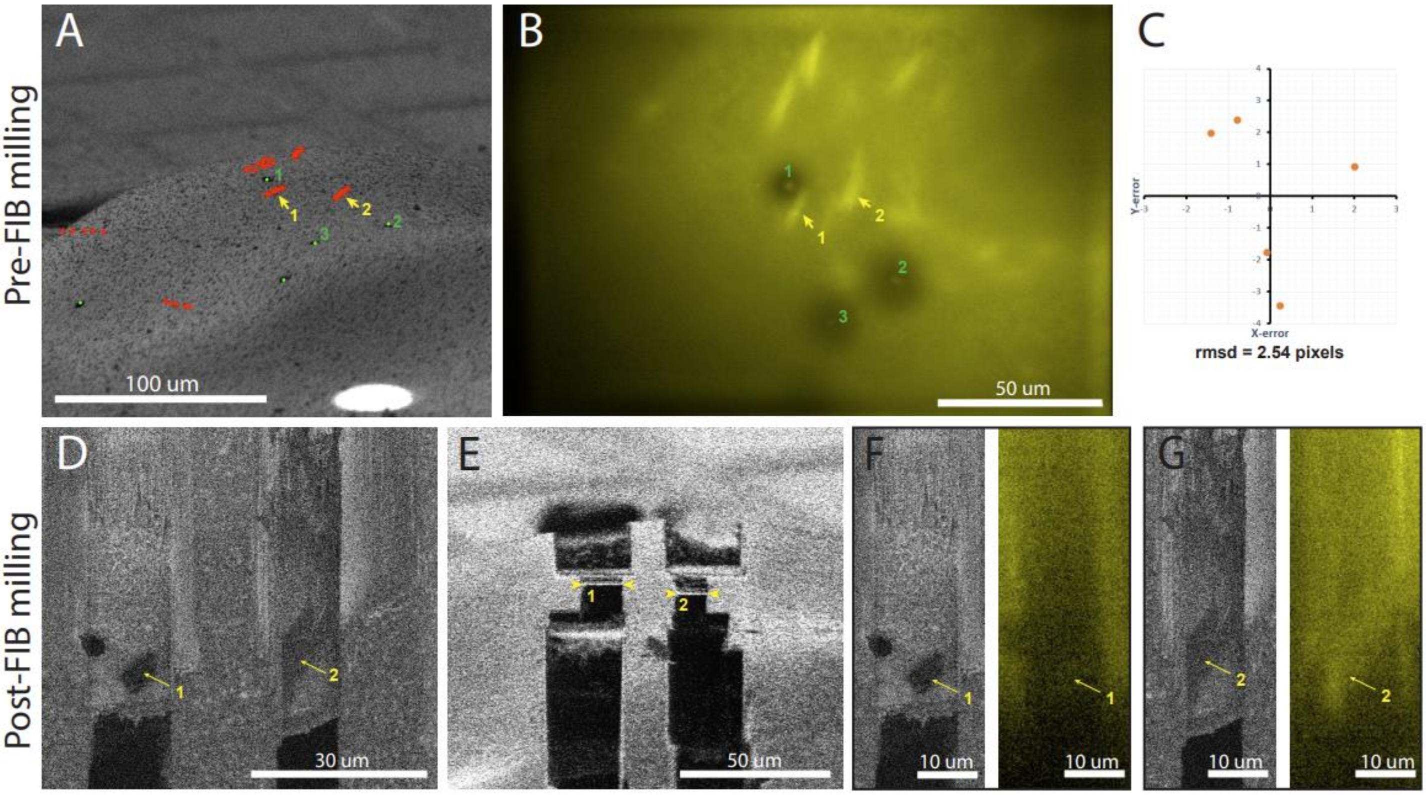

Supplementary Fig. 19. (A) 3DCT correlation of the grazing angle FIB view and the fluorescent stack. Red crosshairs are where the crystals are. Yellow arrows designate the crystals that were targeted for milling and then for micro-ED. Green points show the correlated milled fiducials visible by FIB. (B) iFLM optical slice of the same area of interest. The two same crystals are highlighted with yellow arrows. 3 milled fiducial visible in iFLM (numbered in green) that can also be seen in FIB were highlighted. (C) Scatter plot showing the residual error between the transformed 3D milled fiducial (designated on the fluorescent stack projected on the FIB image) and the designated 2D milled fiducial on the FIB image. The residual square root mean error (RMSE) is 2.54 pixels. (D) SEM image at 1.2kV, in immersion mode, showing the contrast difference of the crystal (yellow arrows) versus the surrounding LCP. (E) FIB view of the final lamellae of the same crystals. (F ang G) Side by side view of the lamella by SEM (left panel) and by fluorescence (right panel).

Supplementary Table 1. Milling steps for each plasma ion beam experiment on proteinase K.

Xenon	Ion-beam current	Box size (x, y, z um)	Pattern type	Time for pattern x2	Pattern separation
Step 1	1.0 nA	6 x 6 x 5	Cleaning cross section (CCS)	5:28	2 um
Step 2	0.3 nA	6 x 1 x 3	CCS	1:52	1 um
Step 3	0.1 nA	5 x 0.5 x 2	CCS	1:34	500 nm
Step 4	30 pA	5 x 0.5 x 1	CCS	2:36	300 nm
Total time milling patterns:				11:30	
Argon	Ion-beam current	Box size (x, y, z um)	Pattern type	Time for pattern x2	Pattern separation
Step 1	2.0 nA	6 x 6 x 6	Cleaning cross section (CCS)	3:16	2 um
Step 2	0.74 nA	6 x 1 x 3	CCS	0:46	1 um
Step 3	0.2 nA	5 x 0.5 x 2	CCS	0:48	500 nm
Step 4	60 pA	5 x 0.5 x 2	CCS	2:36	300 nm
Total time milling patterns:				7:26	
Nitrogen	Ion-beam current	Box size* (x, y, z um)	Pattern type	Time for pattern x2	Pattern separation
Step 1	2.4 nA	6 x 6 x 20	Cleaning cross section (CCS)	9:12	2 um
Step 2	0.78 nA	5 x 1 x 10	CCS	2:04	1 um
Step 3	0.27 nA	5 x 0.5 x 5	CCS	1:32	500 nm
Step 4	47 pA	5 x 0.5 x 5	CCS	8:22	300 nm
Total time milling patterns:				21:20	
Oxygen	Ion-beam current	Box size (x, y, z um)	Pattern type	Time for pattern x2	Pattern separation
Step 1	1.7 nA	6 x 6 x 15	Cleaning cross section (CCS)	8:14	2 um
Step 2	0.61 nA	5 x 1 x 6	CCS	1:06	1 um
Step 3	0.23 nA	5 x 0.5 x 4	CCS	1:06	500 nm
Step 4	90 pA	5 x 0.5 x 4	CCS	3:40	300 nm
Total time milling patterns:				14:06	
Gallium	Ion-beam current	Box size (x, y, z um)	Pattern type	Time for pattern x2	Pattern separation
Step 1	0.5 nA	6 x 6 x 10	Cleaning cross section (CCS)	7:04	2 um
Step 2	0.3 nA	6 x 1 x 6	CCS	1:46	1 um
Step 3	0.1 nA	5 x 0.5 x 3	CCS	1:06	500 nm
Step 4	30 pA	5 x 0.5 x 3	CCS	3:30	300 nm
Total time milling patterns:				13:26	

Supplementary Table 2. Milling steps for each plasma ion beam experiment on $A_{2A}AR$

Xenon	Ion-beam current	Pattern type	Pattern separation
Step 1	4 - 60 nA	Cleaning cross section (CCS)	15 um
Step 2	0.3 nA	CCS	5 um
Step 3	0.1 nA	CCS	1 um
Step 4	30 pA	CCS	300 nm
Step 5	30 pA	Rectangle	300 nm
Argon	Ion-beam current	Pattern type	Pattern separation
Step 1	7.6 - 120 nA	Cleaning cross section (CCS)	15 um
Step 2	0.74 nA	CCS	5 um
Step 3	0.2 nA	CCS	1 um
Step 4	60 pA	CCS	300 nm
Step 5	20 pA	Rectangle	300 nm

Supplementary Table 3. Milling currents for each available ion source on the pFIB

Source	Xenon	Argon	Nitrogen	Oxygen
Aperture #1	1pA	3.9pA	0.58pA	1.3pA
#2	3.0pA	6.0pA	0.64pA	1.4pA
#3	10pA	20pA	1.9pA	4.2pA
#4	30pA	60pA	10pA	20pA
#5	0.1nA	0.2nA	47pA	90pA
#6	0.3nA	0.74nA	0.1nA	0.23nA
#7	1.0nA	2.0nA	0.27nA	0.61nA
#8	4.0nA	7.6nA	0.78nA	1.7nA
#9	15nA	28nA	2.4nA	5.6nA
#10	60nA	0.12uA	23nA	45nA
#11	0.2uA	0.40uA	0.1uA	0.19uA
#12	0.5uA	0.93uA	0.33uA	0.57uA
#13	1.0uA	2.0uA	0.7uA	1.0uA
#14	2.5uA	4.0uA	1.0uA	2.0uA
