# Supplementary material for: A robust approach for MicroED sample preparation of lipidic cubic phase embedded membrane protein crystals
Source: Nat Commun. 2023 Feb 25;14:1086. doi: 10.1038/s41467-023-36733-4 (PMC9968316; doi:10.1038/s41467-023-36733-4)
Supplement: Supplementary file 3 — Reporting Summary [file 41467_2023_36733_MOESM3_ESM.pdf]

Corresponding author(s): Tamir Gonen
Last updated by author(s): Feb 2, 2023

Reporting Summary

Nature Portfolio wishes to improve the reproducibility of the work that we publish. This form provides structure for consistency and transparency in reporting. For further information on Nature Portfolio policies, see our Editorial Policies and the Editorial Policy Checklist.

Statistics

For all statistical analyses, confirm that the following items are present in the figure legend, table legend, main text, or Methods section.

n/a	Confirmed	
☐	☒	The exact sample size (*n*) for each experimental group/condition, given as a discrete number and unit of measurement
☐	☒	A statement on whether measurements were taken from distinct samples or whether the same sample was measured repeatedly
☒	☐	The statistical test(s) used AND whether they are one- or two-sided<br>*Only common tests should be described solely by name; describe more complex techniques in the Methods section.*
☒	☐	A description of all covariates tested
☐	☒	A description of any assumptions or corrections, such as tests of normality and adjustment for multiple comparisons
☐	☒	A full description of the statistical parameters including central tendency (e.g. means) or other basic estimates (e.g. regression coefficient) AND variation (e.g. standard deviation) or associated estimates of uncertainty (e.g. confidence intervals)
☒	☐	For null hypothesis testing, the test statistic (e.g. *F*, *t*, *r*) with confidence intervals, effect sizes, degrees of freedom and *P* value noted<br>*Give P values as exact values whenever suitable.*
☒	☐	For Bayesian analysis, information on the choice of priors and Markov chain Monte Carlo settings
☐	☒	For hierarchical and complex designs, identification of the appropriate level for tests and full reporting of outcomes
☒	☐	Estimates of effect sizes (e.g. Cohen's *d*, Pearson's *r*), indicating how they were calculated

Our web collection on statistics for biologists contains articles on many of the points above.

Software and code

Policy information about availability of computer code

Data collection	VELOX (3.6.0), iFLM (v0.7), TEM (2.6) and SEM (17.21.0) user interface
Data analysis	XDS (VERSION Jan 10, 2022), AIMLESS (0.7.9), CTRUNCATE (1.17.29), PHENIX (1.20.1), REFMAC5 (5.8.0049), FIJI (2.1.0), PHASER (2.8.3), Python/Jupyter (anaconda 3.9), ChimeraX(1.5), Excel (2016), Word (2016), PowerPoint (2016), MAPS (3.21).

For manuscripts utilizing custom algorithms or software that are central to the research but not yet described in published literature, software must be made available to editors and reviewers. We strongly encourage code deposition in a community repository (e.g. GitHub). See the Nature Portfolio guidelines for submitting code & software for further information.

Data

Policy information about availability of data

All manuscripts must include a data availability statement. This statement should provide the following information, where applicable:

- Accession codes, unique identifiers, or web links for publicly available datasets
- A description of any restrictions on data availability
- For clinical datasets or third party data, please ensure that the statement adheres to our policy

All coordinates and structure factors have been deposited to the PDB. All maps have been deposited to the EMDB. Proteinase K accession codes are 8FYP [https://10.2210/pdb8fyp/pdb], 8FYQ [https://10.2210/pdb8fyq/pdb], 8FYS [https://10.2210/pdb8fys/pdb], 8FYR [https://10.2210/pdb8fyr/pdb] for xenon, argon, nitrogen, oxygen, and best merges, respectively. The corresponding EMDB numbers are, in the same order, EMD-29588 [https://www.ebi.ac.uk/pdbe/entry/emdb/

EMD-29588], EMD-29590 [https://www.ebi.ac.uk/pdbe/entry/emdb/EMD-29590], EMD-29596 [https://www.ebi.ac.uk/pdbe/entry/emdb/EMD-29596], EMD-29595 [https://www.ebi.ac.uk/pdbe/entry/emdb/EMD-29595], EMD-29587 [https://www.ebi.ac.uk/pdbe/entry/emdb/EMD-29587]. The coordinates and structure factors for A2AAR are deposited at the PDB with accession code 8FYN [https://10.2210/pdb8fyn/pdb], and the EMDB map codes are EMD-29586 [https://www.ebi.ac.uk/pdbe/entry/emdb/EMD-29586]. All raw data are available from the corresponding author upon request.

Human research participants

Policy information about studies involving human research participants and Sex and Gender in Research.

Reporting on sex and gender	N/A
Population characteristics	N/A
Recruitment	N/A
Ethics oversight	N/A

Note that full information on the approval of the study protocol must also be provided in the manuscript.

Field-specific reporting

Please select the one below that is the best fit for your research. If you are not sure, read the appropriate sections before making your selection.

☒ Life sciences ☐ Behavioural & social sciences ☐ Ecological, evolutionary & environmental sciences

For a reference copy of the document with all sections, see nature.com/documents/nr-reporting-summary-flat.pdf

Life sciences study design

All studies must disclose on these points even when the disclosure is negative.

Sample size	Twenty ideally situated crystals were chosen. These were split evenly between the four possible gases, (n=5 for each beam type).
Data exclusions	Only samples that failed milling were excluded.
Replication	Each gas was tested identically on crystals. Five times each.
Randomization	No randomization was used, as the samples arrange randomly on the grid. Sites were picked and done in order of localization.
Blinding	No blinding was done in this study. Instead, all samples were processed and handled identically during processing.

Reporting for specific materials, systems and methods

We require information from authors about some types of materials, experimental systems and methods used in many studies. Here, indicate whether each material, system or method listed is relevant to your study. If you are not sure if a list item applies to your research, read the appropriate section before selecting a response.

Materials & experimental systems

n/a	Involved in the study	
☒	☐	Antibodies
☒	☐	Eukaryotic cell lines
☒	☐	Palaeontology and archaeology
☒	☐	Animals and other organisms
☒	☐	Clinical data
☒	☐	Dual use research of concern

Methods

n/a	Involved in the study	
☒	☐	ChIP-seq
☒	☐	Flow cytometry
☒	☐	MRI-based neuroimaging
